# Scaling Multimodal Agentic AI in Medical Education: Multisite Cross-Sectional Study of Simulation Effectiveness in Primary Care

**DOI:** 10.2196/88905

**Published:** 2026-03-23

**Authors:** Chris Jacobs, Hans Johnson, Kirsty Brownlie, Richard Joiner, Trevor Thompson

**Affiliations:** 1 Department of Psychology University of Bath Bath, England United Kingdom; 2 Faculty of Life Sciences & Medicine King's College London London, England United Kingdom; 3 Medical School University of Bristol Bristol, England United Kingdom

**Keywords:** artificial intelligence, AI: medical, conversational, education, realism, clinical

## Abstract

**Background:**

Conversational artificial intelligence (AI) systems offer potential solutions to traditional constraints in medical consultation skills training, including high costs, scheduling difficulties, and varied standardization. There is limited evidence evaluating medical professionals’ perceptions of AI-generated patient interactions across multiple fidelity dimensions and assessing the educational value of conversational AI for consultation skills training.

**Objective:**

This study aimed to evaluate perceptions of conversational AI patient simulations in primary care consultation training, examining functional fidelity, conversational realism, educational value, and implementation readiness.

**Methods:**

A cross-sectional evaluation study at a UK medical school (medical students and general practitioners) yielded 47 grouped and individual responses. Participants completed standardized clinical scenarios using the SimFlow conversational AI system, a conversational AI system, followed by a multidomain questionnaire evaluating AI realism, medical content, educational value, feedback, and usability. Data were analyzed using the Wilcoxon signed rank test, Spearman correlation, and Firth logistic regression to assess domain performance and participant characteristics.

**Results:**

Medical content received the highest ratings (median 4.5, IQR 4.0-5.0), with 97.8% (45/46) rating clinical plausibility highly. Educational value was rated positively (median 4.0, IQR 3.0-4.0), although AI realism received moderate scores (median 3.0, IQR 2.0-4.0). Participants with prior AI experience gave significantly higher ratings for AI realism than those without prior experience (mean 3.81, SD 0.63 vs 3.07, SD 0.72; *P*=.03). Concordance analysis demonstrated moderate-to-strong agreement between individual- and group-level domain rankings (mean Spearman ρ=0.685), supporting consistency between collaborative and individual survey evaluations. Qualitative analysis revealed 4 themes: clinical authenticity, interactional limitations, educational potential, and implementation considerations.

**Conclusions:**

Conversational AI demonstrates strong capabilities in functional fidelity (clinical accuracy) despite limitations in conversational fidelity (realism). The technology shows promise as a supplementary tool for clinical skills training rather than higher-stakes assessment, with future development needed in dialogue naturalness and feedback capabilities.

## Introduction

Artificial intelligence (AI) is widely regarded as the suite of technologies that allow computers to perform tasks that resemble human abilities, including pattern recognition, decision-making, and content generation [1]. It has shown considerable promise in health care applications over recent years, with prospects for continued development in medical training contexts [2]. The integration of AI into medical education represents a significant development in health care training approaches, as traditional consultation skills training has long relied on human standardized patients and role-playing exercises. While these conventional methods remain valuable, they face notable constraints, including high costs, scheduling difficulties, and limited standardization across encounters [3].

Large language models (LLMs) and conversational AI systems have demonstrated notable capabilities across health care applications. These systems have shown the ability to pass standardized medical examinations such as the United States Medical Licensing Examination with performance levels approaching human benchmarks [4]. More sophisticated domain-specific models such as Med-PaLM2 have achieved expert-level performance on complex medical questions, indicating their potential for specialized health care applications [5,6]. These capabilities translate effectively into virtual patient simulations, where LLM-sourced avatars can provide authentic and professional patient scenario dialogue flows that enable realistic clinical interactions through large data–resourced models [7]. Multimodal technologies have quickly updated from basic text generation to systems capable of processing voice, text, and visual inputs.

Conversational AI using multimodal agentic systems designed for medical training offer several advantages over traditional methods of simulating patient interactions. They provide continuous availability, enabling students to practice consultation skills without the logistical constraints of coordinating with human actors. These systems can deliver standardized case presentations, ensuring learners encounter consistent scenarios and receive uniform training experiences. The scalability allows multiple simultaneous interactions, addressing resource limitations that have historically restricted access to hands-on practice opportunities [8]. Recent studies have demonstrated the practical application of these technologies, with research showing that medical students can effectively use readily available AI tools such as GPT-4o Advanced Voice Mode for challenging communication skills practice, finding the technology both useful and acceptable for supplementing traditional training methods [9]. Models can be developed for feedback that provides insights into clinical reasoning, language use, and response effectiveness [10]. Furthermore, AI-created feedback offers potential advantages over human supervisor feedback by providing detailed, criterion-based assessments with multiple verbatim quotations from transcribed clinical interactions, enabling personalized learning conversations [11].

Simulation fidelity encompasses multiple dimensions beyond visual and auditory realism, including psychological fidelity and functional fidelity that support specific learning objectives [12]. Understanding these different dimensions of fidelity is important for developing effective AI-based training systems, as research suggests that functional fidelity, the degree to which a simulation evokes targeted cognitive processes, may be more significant for learning outcomes than perfect physical or conversational realism [13]. The conceptual framework for how users interact with immersive technologies encompasses multiple interconnected domains, including engagement, system usability, and cognitive processes, that together influence the overall user experience and educational effectiveness [14].

Recent work has explored how AI can benefit simulation-based training through advanced assessment capabilities and adaptive learning approaches [15,16]. Current research on AI in medical education has primarily focused on knowledge assessment and content generation rather than interpersonal skills training through experiential learning approaches, though systematic reviews indicate growing evidence of AI’s broader educational impact across health professions [17,18]. While studies have examined medical students’ interactions with AI tutoring systems and assessment tools, there is growing recognition of AI’s potential for supporting communication skills training in health care education, though limited research has evaluated how different dimensions of simulation fidelity impact learning outcomes in conversational AI applications for consultation skills development [19]. Training effectiveness and optimizing learning outcomes are becoming apparent, though questions remain about their readiness for widespread implementation [20].

The specific aims of this research were to (1) evaluate how medical professionals perceive the realism and clinical authenticity of AI-generated patient interactions across multiple fidelity dimensions, distinguishing between functional fidelity (clinical accuracy and medical content) and conversational fidelity (realism and flow); (2) assess the educational value of conversational AI for experiential learning in consultation skills training; (3) identify factors that influence user acceptance and perceived simulation effectiveness, particularly regarding the relationship between technical realism and functional educational outcomes; and (4) determine current readiness and implementation considerations for AI-constructed patient simulation in medical education curricula.

## Methods

### Study Design and Participants

We conducted a cross-sectional evaluation study of a conversational AI system designed for patient consultation training. Participants were recruited from a single UK university medical school incorporating 70 general practice sites using a census sampling approach. All medical students in their third year of study and general practitioners within these settings were invited to participate via email invitation during March 2025. Invitations were distributed by administrative education teams and included study information, participation instructions, and survey links. A retrospective protocol for this study, including the full methodology, questionnaire, and analysis plan, was registered on the Open Science Framework prior to journal submission [21].

### Procedure

**Figure 1:**
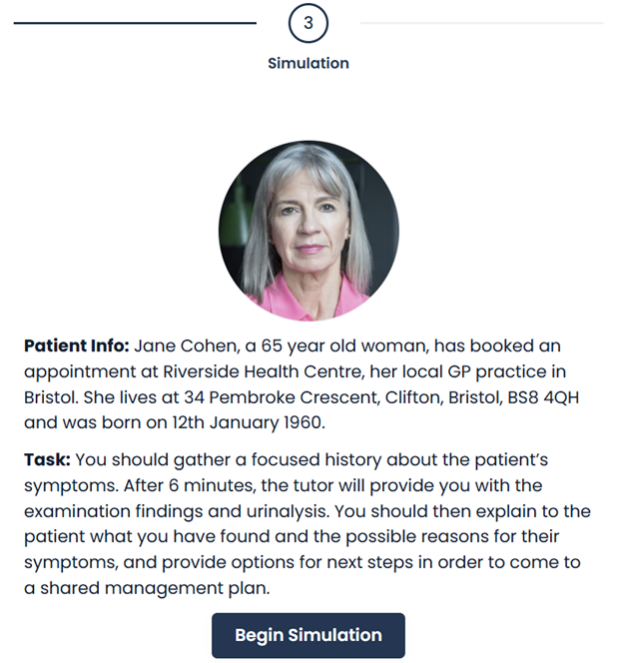
Screenshot of the SimFlow web-hosted conversational artificial intelligence patient simulation platform used during standardized primary care consultation training sessions across UK teaching practices (March 2025).

Participants completed a standardized clinical scenario with a conversational AI system during a scheduled primary care training day. The simulation was delivered through the SimFlow.ai web-hosted platform (Figure 1), which operates within an institutional firewall-protected environment using single-stage authentication. The platform was accessed simultaneously by more than 70 participating teaching practices during a single morning session.

The simulated patient was powered by a proprietary fine-tuned OpenAI GPT model accessed via the OpenAI application programming interface. The deployed model configuration remained constant throughout the evaluation period (February to March 2025). Scenario-specific instructions and application-layer prompt engineering were applied to maintain a consistent simulated patient role and constrain responses within the intended educational context.

Inference parameters were fixed across all users. Temperature (model configuration parameter) was set to 0.7, and maximum response length was configured using a per–simulated patient character baseline cap to support different communication styles (eg, more reserved vs more talkative), with an average maximum of approximately 120 tokens. In addition, the deployment included a sentiment-adaptive dialogue component that monitored conversational cues and modulated response verbosity turn-by-turn within the character’s configured bounds, supporting realistic variability across interactions. The system did not have access to internet browsing or external tools during consultations and relied solely on the current conversation context window acting as memory, with no external retrieval.

A synthetic voice engine generated an age-appropriate female voice matched to the demographic profile of the virtual patient. The AI operated in a one-way configuration in which user inputs were not stored or transmitted externally beyond the secure hosting environment. All sites undertook the same scenario, involving a primary care consultation with a woman in her 60s presenting with a first episode of visible hematuria. Learners were instructed to conduct the consultation as they would in routine clinical practice, including eliciting the history, identifying relevant red flag symptoms, and discussing appropriate investigation and management options.

The underlying prompt design was developed from a summarized version of the case-based learning materials used in the teaching session and mapped closely to the intended learning outcomes. This ensured that the virtual patient’s history, symptom cues, and conversational structure aligned with established clinical reasoning expectations and supported the educational aims of the activity.

Prior to deployment, the scenario underwent face validity testing with practicing clinicians, medical educators, and individuals experienced in simulation design. Feedback from this process informed iterative refinements to the prompt, including clearer symptom cues, improved clinical accuracy, and more coherent conversational flow. Supporting materials for students and facilitators, including case notes, learning points, and debriefing guidance, were also reviewed for accuracy and curricular alignment.

The simulation was delivered in a facilitated training setting. Members of the academic team were present across participating sites remotely to support implementation and oversight. The simulated patient was constrained via scenario- and role-specific prompts, including separate guardrail instructions, to ensure responses remained within the simulation context. Outputs were monitored during the session, and transcripts were reviewable post hoc, enabling sessions to be paused and reviewed if required. No safety concerns were reported during deployment, and no clinically inappropriate outputs were observed during facilitator observation or post hoc transcript review.

Platform safeguards included role-based access controls, audit logging, and encryption in transit and at rest. OpenAI was used as a subprocessor under contractual safeguards, and user data were not used for model training within the service.

### Data Collection

Following the consultation, participants completed a structured questionnaire evaluating the system across 5 domains (Multimedia Appendix 1): AI realism (6 items), medical content (2 items), educational value (3 items), feedback (2 items), and usability (2 items). Each item was rated on a 5-point Likert scale (1=strongly disagree to 5=strongly agree). A total of 3 open-ended questions captured qualitative feedback on the system’s most realistic aspects, areas for improvement, and suitability for assessment purposes such as objective structured clinical examinations. Domains relating to engagement and system usability abridged from immersive technology in medical education measure (Immersive Technology Evaluation Measure) [22]. Furthermore, the working group pilot-tested for construct validity check. The overall questionnaire demonstrated excellent internal consistency (Cronbach α=0.92). Factor analysis supported the 5-domain structure, with internal consistency ranging from good (user experience: α=0.753) to excellent (feedback: Cronbach α=0.954 and learning value: Cronbach α=0.935), confirming the validity of the theoretical framework for evaluating conversational AI acceptability in medical education. Survey methods are reported using the Checklist for Reporting of Survey Studies checklist [23] (Multimedia Appendix).

Demographic information collected included professional role, years of clinical experience, and prior experience with conversational AI systems for medical training.

### Data Analysis Plan

Quantitative data analysis was performed on 47 unique questionnaire responses, representing a mix of individual submissions and group consensus submissions. The unit of analysis was defined a priori as the completed survey instrument rather than the individual respondent. Surveys were completed either by individual participants or collaboratively by groups; where surveys were completed jointly, each group-completed survey was treated as a single data point. This approach reflects the level at which data were generated and avoids inappropriate assumptions of independence among contributors to group responses.

As this was an exploratory feasibility study, aggregated group responses were retained for analysis. The implications of this analytic decision, including limitations related to inference at the individual level, are acknowledged in the Discussion section.

Data cleaning and statistical analysis were conducted using R statistical software (R Foundation for Statistical Computing). Internal consistency of the questionnaire domains was assessed using Cronbach α. One-sample Wilcoxon signed-rank tests were used to determine if domain scores differed significantly from a neutral median of 3.0. Mann-Whitney *U* tests were used to evaluate subgroup differences based on professional role and prior AI experience. For odds ratio (OR) calculations comparing high ratings (≥4) between groups with and without prior AI experience, we used Firth penalized logistic regression. All statistical tests were 2-tailed, and a *P* value <.05 was considered statistically significant.

Qualitative analysis of open-ended survey responses was conducted using reflexive thematic analysis following the framework of Braun and Clarke [24]. A total of 2 researchers independently familiarized themselves with the data and generated initial codes. These codes were iteratively reviewed and collated into overarching themes regarding clinical authenticity, interactional limitations, and educational potential. Qualitative findings were triangulated with quantitative results to provide context for statistical trends, specifically regarding the divergence between medical content accuracy and conversational realism.

### Patient and Public Involvement

Health care professionals and students were interviewed in the design process of the Immersive Technology Evaluation Measure survey [14,25]. During the evaluation, patients in 1 primary care practice were asked for their views on this training method. No objections were raised regarding the use of agentic AI to simulate conversations. Medical actors (n=34) were consulted about using AI in simulation, with 30 (88%) expressing a positive attitude toward involvement in future research on agentic AI.

### Ethical Considerations

This study was approved by the Swindon Academy Medical Education and Ethics Committee Institutional Review Board (CJ062023). All participants provided voluntary informed consent after receiving a comprehensive explanation of the study. Data were anonymized, deidentified, and stored on a secure server. No compensation was provided for participating in the study.

## Results

### Participant Characteristics

From the eligible population of 305 health care professionals (n=245, 80.3% medical students and n=60, 19.7% general practitioners), the adjusted response rate was 61.3% (n=179). The final sample included evaluations from medical students (27/47, 57.4%), general practitioners (18/47, 38.3%), and specialist doctors (1/47, 2.1%). Most participants had clinical experience of either 1 to 2 years (17/47, 36.2%) or >11 years (14/47, 29.8%), with fewer in intermediate experience categories (3-5 years: 10/47, 21.3% and 5-10 years: 6/47, 12.8%). Only 12.8% (6/47) reported prior experience with conversational AI for medical training.

### Quantitative Findings

#### Domain-Level Evaluation

**Figure 2:**
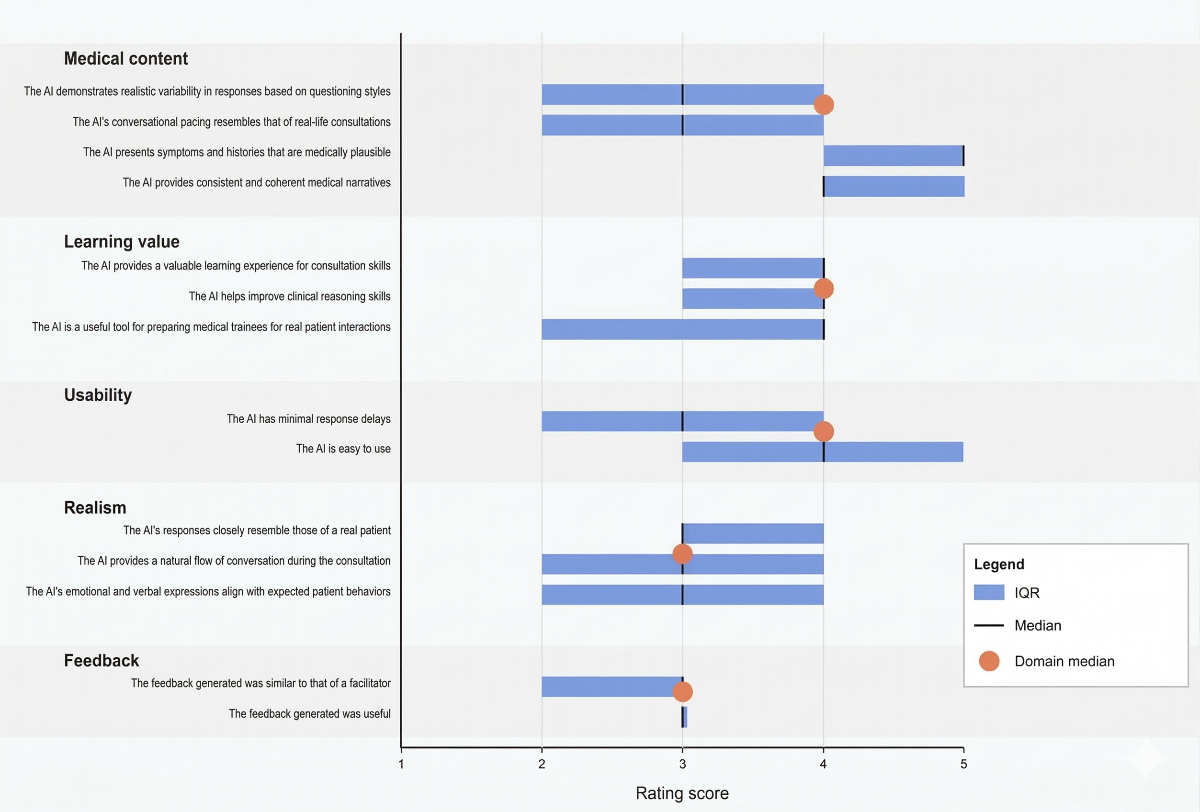
Domain-level participant ratings of conversational artificial intelligence (AI) simulation across fidelity and usability domains among medical students and general practitioners following participation in a standardized hematuria consultation scenario in UK primary care training settings (March 2025).

Table 1 presents descriptive statistics for the 5 evaluation domains (additionally in Figure 2). Medical content received the highest ratings (median 4.5, IQR 4.0-5.0), with nearly all participants (45/46, 97.8%) rating the medical plausibility of the AI system highly. Educational value was also rated positively (median 4.0, IQR 3.0-4.0), though with greater variability in responses. AI realism received moderate ratings (median 3.0, IQR 2.0-4.0), with voice quality (median 4.0) scoring higher than other realism aspects (median 3.0). Feedback capabilities received the lowest consistent ratings (median 3.0, IQR 2.0-3.0).

**Table 1:** Domain-level participant ratings of conversational artificial intelligence (AI) patient simulation across fidelity, usability, and educational value domains among medical students and general practitioners following completion of a standardized hematuria consultation scenario in UK primary care teaching practices (March 2025).

Domain	Value, median (IQR)	Value, range
Medical content	4.0 (4.0-5.0)	3-5
Learning value	4.0 (3.0-4.0)	1-5
User experience (usability)	4.0 (3.0-5.0)	2-5
AI realism	3.0 (2.0-4.0)	1-5
Feedback	3.0 (2.0-3.0)	1-5

**Figure 3:**
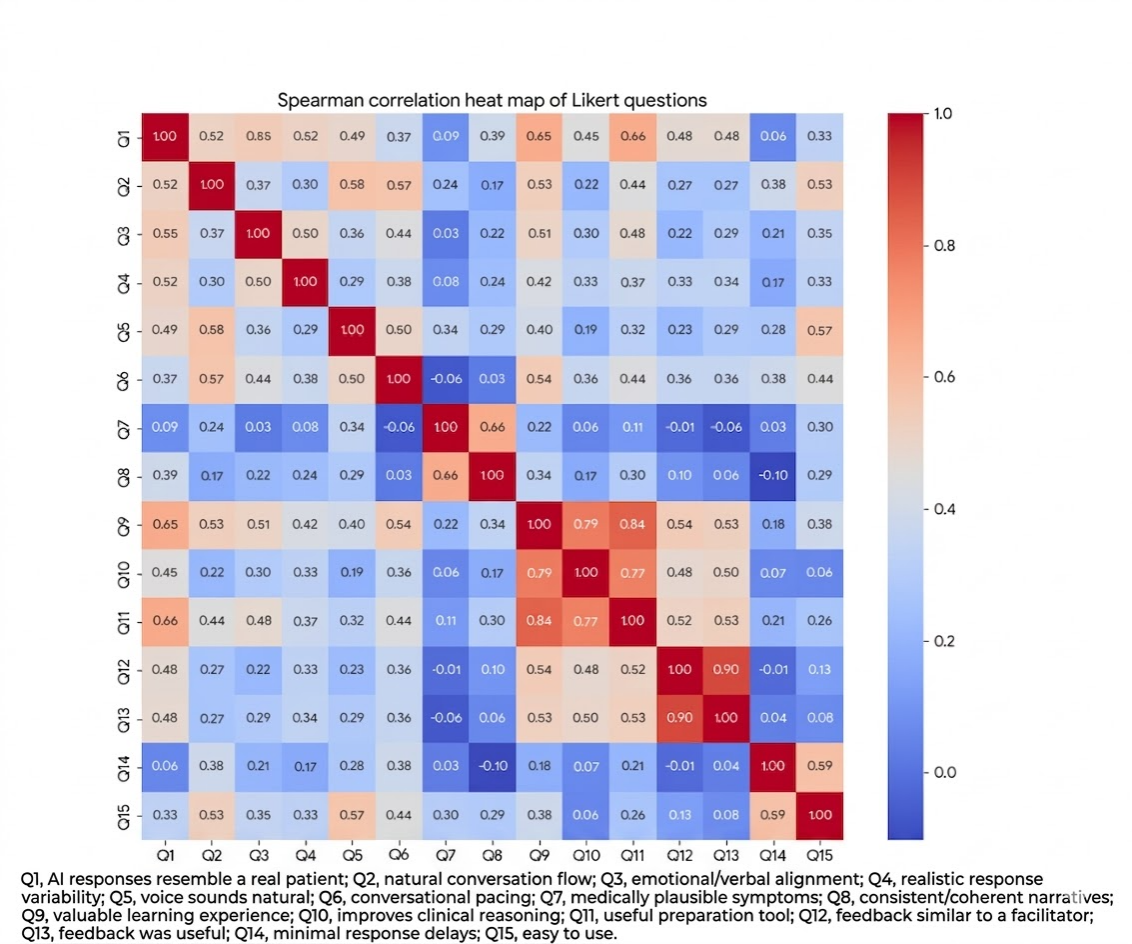
Heat map demonstrating correlation strength between questionnaire items evaluating conversational artificial intelligence simulation fidelity, usability, and educational value among medical students and general practitioners following participation in a standardized hematuria consultation training scenario in UK primary care teaching settings (March 2025).

On the basis of individual-level concordance analysis, 46.8% (22/47) of participants demonstrated high or very high agreement with the group findings (≥3 exact domain rank matches), with an additional 25.5% (17/47) showing moderate concordance (2 exact matches), and an average Spearman correlation of 0.685 (moderate-to-strong agreement; Figure 3).

#### Effect of Participant Characteristics

**Figure 4:**
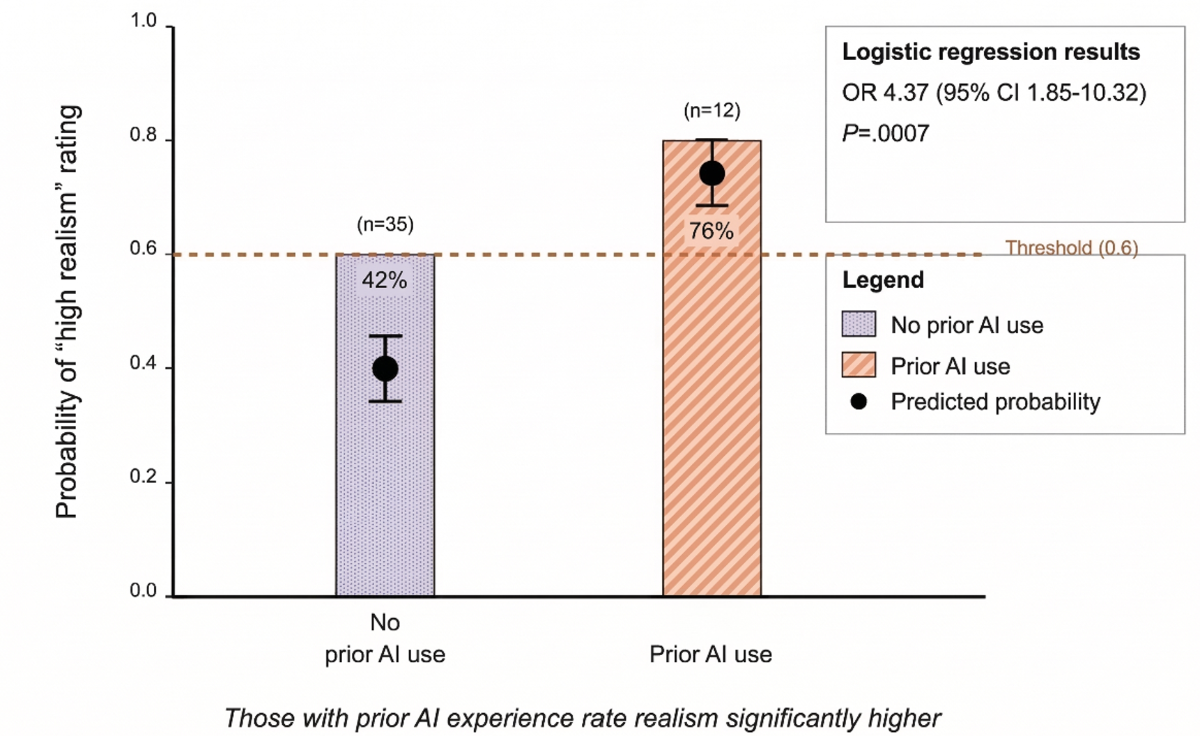
Logistic regression model demonstrating association between prior conversational artificial intelligence (AI) experience and probability of assigning high realism ratings to conversational AI patient simulation among medical students and general practitioners participating in a standardized hematuria consultation scenario in UK primary care training settings (March 2025). OR: odds ratio.

Prior AI experience (*expectation effect*) was associated with higher ratings for AI realism when analyzing mean scores (with experience: 3.81, SD 0.63; without experience: 3.07, SD 0.72; difference: 0.74; *t*_7_=2.63; *P*=.03), as seen in Figure 4. In the OR analysis, participants with prior AI experience were more likely to give high ratings (≥4) for AI realism (OR 3.69, 95% CI 0.54-21.89) and user experience (OR 2.29, 95% CI 0.45-14.35), though these CIs included 1.0 (Table 2). For medical content, where near-ceiling effects were observed (100% vs 97.6% with high ratings), the OR was 0.48 (95% CI 0.02-73.37), with the wide CI reflecting the small sample size among participants with prior AI experience and limited variability in this domain.

**Table 2:** Impact of prior artificial intelligence (AI) experience on domain scores among participants evaluating conversational AI patient simulation during a standardized primary care hematuria consultation training exercise in UK teaching practices (March 2025).

Domain	High ratings with AI experience (n=6), n (%)	High ratings without AI experience (n=41), n (%)	Odds ratio^a^ (95% CI)
AI realism	2 (33.3)	5 (12.2)	3.60 (0.52-21.89)
User experience	4 (66.7)	18 (43.9)	2.56 (0.42-14.35)
Educational value	3 (50.0)	20 (48.8)	1.05 (0.19-5.50)
Feedback	1 (16.7)	7 (17.1)	0.97 (0.10-9.65)
Medical content	6 (100.0)	40 (97.6)	0.30 (0.02-73.37)

^a^Odds ratios derived using Firth penalized logistic regression.

#### Question-Level Analysis

The highest-rated individual item was “The AI presents symptoms and histories that are medically plausible” (median 5.0, IQR 4.0-5.0), followed by “The AI provides consistent and coherent medical narratives” (median 4.0, IQR 4.0-5.0). “Is easy to use” also received high ratings (median 4.0, IQR 3.0-5.0). The lowest-rated items were “The feedback generated was similar to facilitator” (median 3.0, IQR 2.0-3.0) and “has minimal response delays” (median 3.0, IQR 2.0-4.0). Table 3 and Figure 2 summarize these item results against a neutral baseline.

**Table 3:** Item-level participant ratings of conversational artificial intelligence (AI) simulation fidelity, usability, and educational utility following participation in a standardized hematuria consultation scenario in UK primary care teaching settings (March 2025)a.

Domain or item		Value, median (IQR)	*P* value
**Medical content (functional fidelity)**			
	Medically plausible symptoms (Q7^b^)	5.0 (4.0-5.0)	<.001
	Consistent or coherent narratives (Q8^c^)	4.0 (4.0-5.0)	<.001
**AI realism (conversational fidelity)**			
	Voice sounds natural (Q5^d^)	4.0 (3.0-4.0)	.001
	Responses resemble a real patient (Q1^e^)	3.0 (3.0-4.0)	.43
	Natural flow of conversation (Q2^f^)	3.0 (2.0-4.0)	.76
	Emotional or verbal alignment (Q3^g^)	3.0 (2.0-4.0)	.58
	Realistic response variability (Q4^h^)	3.0 (2.5-4.0)	.33
	Conversational pacing (Q6^i^)	3.0 (2.0-4.0)	.89
**Educational value**			
	Valuable learning experience (Q9^j^)	4.0 (3.0-4.0)	.01
	Improves clinical reasoning (Q10^k^)	4.0 (3.0-4.0)	.02
	Useful preparation tool (Q11^l^)	4.0 (2.0-4.0)	.11
**Feedback and usability**			
	Easy to use (Q15^m^)	4.0 (3.5-5.0)	<.001
	Feedback similar to a facilitator (Q12^n^)	3.0 (2.25-3.0)	.17
	Feedback was useful (Q13^o^)	3.0 (3.0-3.0)	.21

^a^Statistical significance determined using 1-sample Wilcoxon signed-rank testing against a neutral midpoint rating of 3.0.

^b^Q7: medically plausible symptoms.

^c^Q8: consistent or coherent narratives.

^d^Q5: voice sounds natural.

^e^Q1: AI responses resemble a real patient.

^f^Q2: natural conversation flow.

^g^Q3: emotional or verbal alignment.

^h^Q4: realistic response variability.

^i^Q6: conversational pacing.

^j^Q9: valuable learning experience.

^k^Q10: improves clinical reasoning.

^l^Q11: useful preparation tool.

^m^Q15: easy to use.

^n^Q12: feedback similar to a facilitator.

^o^Q13: feedback was useful.

### Qualitative Findings

**Figure 5:**
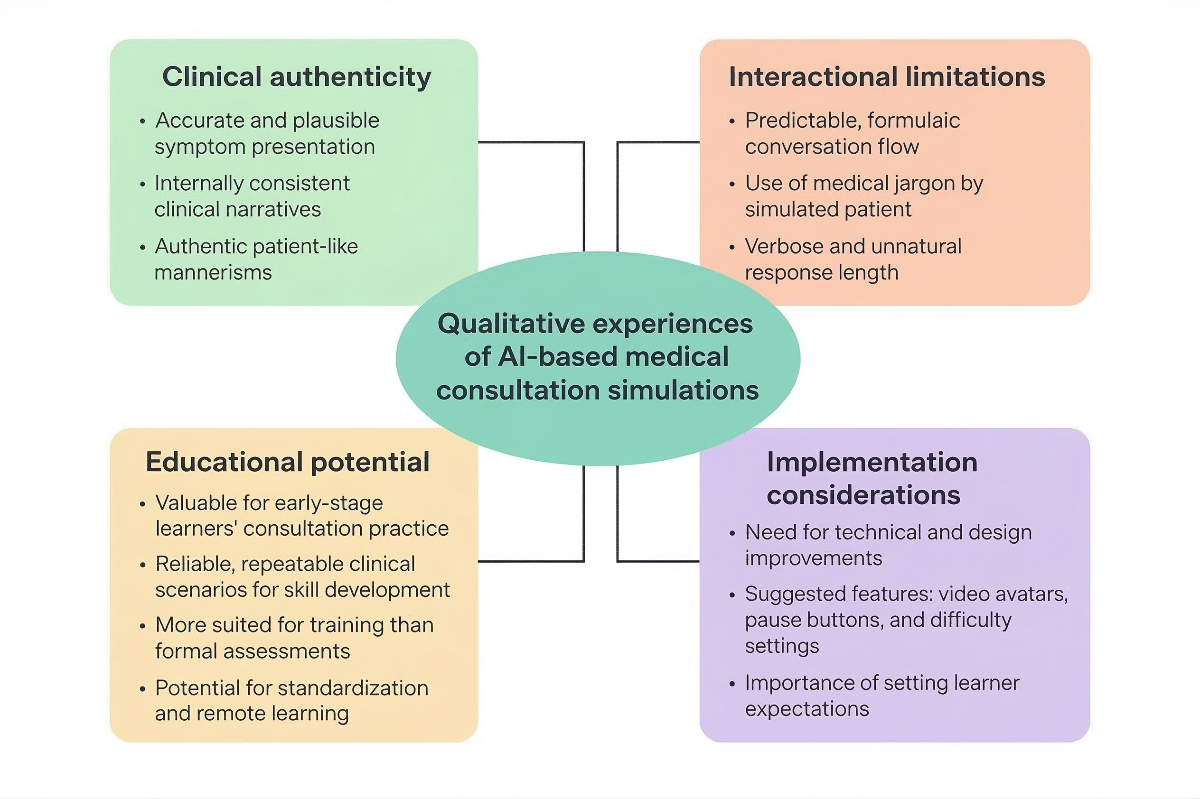
Thematic map illustrating qualitative themes identified from participant feedback regarding conversational artificial intelligence (AI) patient simulation following completion of a standardized hematuria consultation scenario in UK primary care teaching settings (March 2025).

Thematic analysis of open-ended responses revealed 4 key themes: clinical authenticity, interactional limitations, educational potential, and implementation considerations. Figure 5 summarizes the key themes and subthemes related to response.

#### Clinical Authenticity

This clinical reliability was frequently noted as the system’s greatest strength, even when other aspects were criticized:

Mannerisms sounded human, plausible responses, given good history. Responding well to questions asked. Patient sounded appreciative and positive.

Participant #18

Many participants observed that the authenticity of clinical content partially compensated for conversational limitations, suggesting that strong medical accuracy might be more important than perfect conversational flow for educational applications.

#### Interactional Limitations

Participants frequently identified unnatural conversational patterns that diminished the realism of the consultation experience:

The AI answered almost every question with a question which disrupted the flow of the consultation. Almost every answer was followed by “is that what you’d expect”/“does that rule anything out” which became a bit repetitive and did not feel natural.

Participant #1

#### Educational Potential

Despite limitations, participants recognized significant training value, particularly for early-stage learners who need practice with basic consultation skills and clinical reasoning before engaging with real patients. The system’s ability to provide consistent scenarios with reliable clinical presentations was seen as valuable for skill development:

Consultation skills—her answers were life like, as if talking to a real patient.

Participant #25

#### Implementation Considerations

Participants identified specific technical and design improvements needed before wider deployment, focusing on both technological limitations and pedagogical design elements that would enhance the educational experience:

1. Monologue 2. Information given too readily 3. Knew what question would be 4. Less prompt based and more response based.

Participant #22

### Triangulation of Findings

Quantitative and qualitative findings showed strong convergence. The high ratings for medical content aligned with qualitative praise for clinical plausibility. Similarly, the moderate ratings for conversational aspects corresponded with qualitative feedback about unnatural dialogue patterns. The statistically significant difference showing that participants with prior AI experience gave higher realism ratings (*t*₇=2.63; *P*=.03) aligns with qualitative comments suggesting expectations influence perception of the system’s capabilities.

## Discussion

### Principal Findings

The system demonstrated high functional fidelity, with “medical plausibility” scoring significantly above the neutral baseline (median 5.0; *P*<.001). This confirms that current LLM-based simulations can effectively replicate the “medical logic” of a patient encounter. However, conversational fidelity (realism) remained statistically neutral (median 3.0). Participants did not actively reject the realism but perceived it as average, supporting the hypothesis that while the voice was excellent (median 4.0), the emotional and conversational flow lagged behind.

Qualitative analysis identified 4 key themes: clinical authenticity, interactional limitations, educational potential, and implementation considerations. Participants consistently praised the system’s medical knowledge base and clinically accurate presentations, which partially compensated for conversational limitations.

Notably, prior experience with AI technology significantly influenced perception of system realism (*P*<.05), with experienced users rating realism higher (mean 3.60) than those without prior exposure (mean 3.07), highlighting a potential expectation effect.

### Comparison With Other Studies

Our findings align with previous research on AI virtual patient simulation in health care education. Systematic reviews of LLM applications in medical training have similarly found that clinical accuracy and educational utility were rated more favorably than conversational ability in relation to human interaction [26]. The challenge of creating natural dialogue patterns in AI systems has been identified as a technical hurdle despite advances in foundation models [5,27].

The high ratings for medical content accuracy in our study correspond with findings from performance analyses of AI in adopting large datasets, which have documented capabilities in representing detailed information while highlighting limitations in naturalistic conversation [28]. Our findings regarding the high ratings for clinical content accuracy despite lower ratings for conversational realism reinforce important principles about simulation fidelity in health care education. Effective simulation does not require faithful replication of reality but rather accurate representation of essential cues and stimuli [12]. In our study, participants clearly distinguished between clinical plausibility and conversational authenticity, however, they still found significant educational value in the AI simulation. This supports that learning outcomes can be achieved without perfect fidelity in all dimensions [29,30]. Aspects of fidelity significantly hinge on the learners’ perceived realism of the context of the learning episode as opposed to any one particular element.

LLM can perpetuate and amplify existing biases in datasets, which may explain our participants’ observations of the AI’s tendency toward medically precise but conversationally unnatural responses. Research shows that LLMs are primarily optimized for generating accurate informational content rather than simulating natural human conversation patterns [31]. This optimization bias helps explain our qualitative findings, in which participants noted that the AI system often answered questions with questions and produced verbose responses when simpler answers would have been more natural. This tendency to provide excessively detailed information reflects what has been described as a fundamental characteristic of current LLMs, which prioritize comprehensive information delivery over conversational naturalness. For medical education specifically, our participants’ tolerance of these conversational limitations suggests that the educational value of accurate clinical content may outweigh the need for perfect conversational simulation. In a pilot study, similar conversational limitations in their virtual patient system were noted, including unusually formal language, repetitive answers, excessively long responses, and consistently polite demeanor. Latency is an ongoing limitation with current AI technology that incorporates humanlike voice [32], which is due to multiple systems working in tandem to provide the necessary computational steps from text transcription to voice creation [33,34].

There has been significant progress in the ability of LLM to accurately assess a wide range of clinical material [35-37]. Furthermore, the computational capacity exists to analyze the conversation transcription against a chosen framework or rubric [38]. Our findings on conversational AI feedback capabilities align with research on AI-generated educational feedback. In a study using a custom prompt for GPT-4 demonstrated how LLMs can be specifically tailored to educational objectives when guided by established pedagogical frameworks [39]. Similar to our results showing moderate feedback ratings (median 3.0, IQR 2.0-3.0), their study revealed variability in AI performance across different feedback dimensions. This suggests that while current AI systems can successfully incorporate elements of effective feedback principles, they still require refinement in consistently providing high-quality information about learner performance.

The “expectation effect” we observed, where prior AI experience influenced system perception, can be interpreted as technological readiness [40]. This phenomena may have influenced the perception of the conversation AI. In particular, our findings suggest improved realism with prior experience. Users with prior AI exposure develop more realistic expectations about system capabilities and limitations, leading them to evaluate performance relative to known technological constraints rather than human interaction standards [30]. Given the cross-sectional nature of the study and the potential influence of unmeasured factors such as digital literacy or baseline attitudes toward AI, these findings should be interpreted as associative and hypothesis generating.

### Implications for Training

Our findings suggest several tentative implications for medical education. Conversational AI systems may have a role as supplementary, low-stakes tools for early-stage communication skills practice, offering consistent and accessible rehearsal opportunities prior to, or alongside, patient contact. While ratings for medical content and educational value were favorable, these should be interpreted in the context of moderate realism and limitations in conversational flow, indicating that current systems are not substitutes for human interaction.

For medical educators, these systems may partially mitigate challenges in standardizing training experiences and providing equitable access to practice opportunities, particularly in settings with limited standardized patient resources or during disruptions to in-person teaching [3]. The ability to deploy multiple simultaneous interfaces (>70 in this study) demonstrates technical scalability, though its educational effectiveness at scale requires further evaluation before broader curricular adoption [41].

Content may compensate for realism despite the neutral ratings for conversational flow; the educational value remained significantly positive (median 4.0, IQR 3.0-4.0). This supports the theory that functional fidelity (the accuracy of the case) is more critical for clinical reasoning training than conversational fidelity (perfect chat flow). Learners appear willing to tolerate some *robotic* conversational traits provided that the medical signs, symptoms, and history are robust.

However, participants’ distinction between the system’s utility for practice vs assessment (noted in qualitative feedback) suggests caution in higher-stakes implementation. Recent commentary on AI assessment tools in health care education emphasizes that technology-enhanced learning tools should complement human instruction and assessment as an adjunct rather than replace them [42]. Transparency and disclosure of the fundamental nature of AI interactions help avoid misrepresentation of technological capabilities and cultivate an ethical approach and awareness of system biases [43].

### Strengths

The study’s unique methodology allowed for simultaneous testing with more than 70 AI interfaces in a single time window, demonstrating the system’s scalability for large group teaching sessions.

The study also achieved a relatively high adjusted response rate (179/292, 61.3%), suggesting good engagement with the evaluation process [44]. The systematic evaluation across multiple domains using a structured questionnaire built upon validated measures provides a reliable framework for future studies in this area.

### Limitations and Future Research

Several limitations warrant consideration. First, the study was conducted at a single institution that potentially limits generalizability. The uneven distribution of clinical experience among participants (bimodal distribution toward either 1-2 years or >11 years) may have influenced overall ratings. Importantly, surveys completed collaboratively may reflect group dynamics such as dominant voices, social desirability, or facilitation effects; although these responses were analyzed as single data points to avoid nonindependence, they may not be fully equivalent to individually completed surveys, and this could have influenced domain scores and subgroup comparisons. The cross-sectional design provides only a snapshot evaluation rather than longitudinal assessment of the technology’s impact on learning outcomes or skill development.

The design and evaluation of AI-driven virtual platforms that focus on communication skills would benefit from greater stakeholder participation throughout the development process. Including input from diverse user groups, subject matter experts, educators, and industry professionals can provide valuable perspectives that enhance the relevance and effectiveness of these systems. Educational effectiveness can be explored with controlled studies that expand from co-designed learning approach. This collaboration ensures that the resulting platforms address actual communication challenges while incorporating real-world contexts and expectations that might otherwise be overlooked in purely technical development environments [8].

Building on the findings and these methodological limitations identified in this study, our research group is undertaking a prospectively registered program of work to further evaluate conversational AI–based simulation using analytically robust, prespecified methods [45]. The protocol accounts for the structure of simulation-based education, in which learners contribute repeated responses across multiple clinical scenarios and simulation modalities. Quantitative outcomes will be summarized with group-level comparisons (eg, AI-simulated patients vs actor-based simulation; medical students vs general practitioner educators). To address repeated measures and clustering, mixed-effects models were specified a priori, incorporating identifiers as random intercept and adjusting for station or scenario as a fixed effect. This modeling strategy is intended to preserve within-participant correlation, reduce bias arising from clustered educational delivery, and allow more precise attribution of observed differences to simulation modality rather than contextual variation. By embedding this statistical analysis plan within a prospectively defined research protocol, , the study aims to address the limitations of this study, improve reproducibility, and support scalable multisite evaluation of conversational AI in medical education.

### Conclusions

This evaluation demonstrates that conversational AI systems for patient consultation skills training show promising capabilities in providing clinically accurate scenarios with educational value, despite limitations in conversational authenticity. The technology appears particularly suited for supplementary training in clinical skills development rather than high-stakes assessment. Future refinements for large-scale randomized studies should focus on enhancing dialogue naturalness and feedback capabilities while maintaining the strong medical content accuracy that participants valued most highly.

## Appendix

Multimedia Appendix 1 Anonymized data.

## Abbreviations

AI: artificial intelligence
LLM: large language model
OR: odds ratio

## Data Availability

Anonymized data are in the Multimedia Appendix 1.
